# Phenotypic and genotypic analysis of biofilm production by *Pseudomonas aeruginosa* isolates from infection and colonization samples

**DOI:** 10.1590/0037-8682-0399-2020

**Published:** 2020-10-21

**Authors:** Rodrigo Lira Rodrigues, Jailton Lobo da Costa Lima, Kêsia Xisto da Fonseca Ribeiro de Sena, Maria Amélia Vieira Maciel

**Affiliations:** 1 Universidade Federal de Pernambuco, Centro de Ciências Médicas, Coordenação de Área Medicina Tropical, Recife, PE, Brasil.; 2 Universidade Federal de Pernambuco, Centro de Biociências, Departamento de Antibióticos, Recife, PE, Brasil.

**Keywords:** Biofilm, Colonization, Pseudomonas aeruginosa, Quorum sensing, Surveillance

## Abstract

**INTRODUCTION:**

*Pseudomonas aeruginosa* is an opportunistic pathogen associated with healthcare-related infections, affecting mainly patients with underlying diseases and immunosuppression. This microorganism has several virulence mechanisms that favour its pathogenesis, including the production of biofilm. This study aimed to analyze the phenotypic production of biofilms, the occurrence of quorum sensing (QS) genes, and the clonal profile of clinical isolates of *P. aeruginosa* from colonized/infected patients in a tertiary hospital in Recife-PE.

**METHODS:**

We obtained 21 isolates that were classified as infection isolates (II), and 10 colonization isolates (CI). The phenotypic analysis for biofilm production was performed quantitatively. The QS genes were detected by specific PCRs, and the clonal profile was assessed using ERIC-PCR.

**RESULTS:**

Of the 31 isolates, 58.1 % (18/31) were biofilm producers, of which 70 % (7/10) were CI and classified as weakly adherent; 52.4 % (11/21) of the II produced biofilms, and were classified as weak (38.1 %, (8/21)), moderate (9.5 %, (2/21)), and strongly adherent (4.8 %, (1/21)). All isolates harbored the QS genes analyzed. In the clonal analysis, 26 distinct genetic profiles were identified, highlighting the presence of a clone in four samples, i.e., one infection isolate, and 3 colonization isolates.

**CONCLUSIONS:**

The detection of biofilm formation is important in *P. aeruginosa* in addition to the identification of colonization and infection isolates, especially from complex environments such as ICUs. Further, we define a strategy for monitoring and analyzing *P. aeruginosa* strains that can potentially cause infections in hospitalized patients.

## INTRODUCTION

*Pseudomonas aeruginosa* is a bacillus species found in soil, water, and the digestive tract of some animals. It is grown in the laboratory on cetrimide agar which is used for the selective isolation of bacteria, and it enables the observation of the large, mucoid, greenish colonies that have a characteristically sweetish odor and fluoresce in the presence of ultraviolet light due to the production of pyoverdin. Additionally, the colonies have been shown to withstand temperatures of 4 - 42ºC^1^ ^,^ ^2^.

The *P. aeruginosa* bacterium is an opportunistic pathogen that colonizes immunosuppressed individuals that have underlying diseases, and patients who are admitted to the intensive care units (ICU) in hospitals^2^ ^,^ ^3^ ^,^ ^4^. Due to its virulence, the pathogen can colonize second and third-degree burns in individuals, patients on mechanical ventilation, biomaterials (such as catheters, prostheses, and contact lenses), the urinary tract, and the cornea^5^ ^,^ ^6^

*P. aeruginosa* is an important etiological agent associated with healthcare-related infections and it has been shown to increase the rate of mortality and morbidity in patients. It can potentially become multidrug-resistant due to its ability to acquire different antimicrobial resistance mechanisms^3^ ^,^ ^6^ ^,^ ^7^ ^,^ ^8^ ^,^ ^9^.

The pathogen shows the ability to produce biofilms, which are an important factor for virulence and bacterial resistance, and can have a strong impact on the health of the host. The dense polysaccharide matrix of the biofilm contributes to the persistence of infection, the ineffective action of antimicrobials, and the escape from the phagocytic actions of the cells of the immune system of the host, these effects result in chronic pathology^5^ ^,^ ^6^ ^,^ ^10^ ^,^ ^11^. Biofilms enable the survival of bacteria on abiotic surfaces and facilitate pathogen permanence and its dissemination between hospital beds^8^ ^,^ ^12^ ^,^ ^13^. An estimated 80% of *P. aeruginosa* infections are associated with biofilm production, such as in patients undergoing mechanical ventilation, or those having cystic fibrosis or burns^3^ ^,^ ^5^ ^,^ ^6^ ^,^ ^14^.

Like mammalian cells, bacteria respond to molecules and interact with the receptors of other microorganisms to communicate^1^ ^,^ ^15^. This biochemical interaction is necessary for the formation of biofilms and the production of other virulence factors. These molecules, which are self-inducting, can be produced and secreted by the bacterial community itself. At high concentrations, these molecules stimulate biofilm production due to the transcription of specific genes. This complex communication mechanism is called quorum sensing (QS)^13^ ^,^ ^16^ ^,^ ^17^. QS enables *P. aeruginosa* to respond to the self-inducting molecules called acyl-homoserine lactones (AHLs) that target the *Las* and *Rhl* genes. 

Further, the *lasI, rhlI*, and *pqsA* genes are responsible for the biosynthesis of the signaling molecules N-(3-oxododecanoyl)-L-homoserine lactone (OdDHL), N-Butyrylhomoserine lactone (BHL), and the *Pseudomonas* quinolone signal (PQS), respectively. The corresponding receptors for the signaling molecules are LasR, RhlR, and PqsR, respectively, which are involved in the regulation of bacterial virulence and biofilm formation^17^ ^,^ ^18^ ^,^ ^19^ ^,^ ^20^.

In this study, we aimed at analyzing *P. aeruginosa* isolates from colonized and infected patients to verify biofilm production phenotypically, search for QS genes (*lasI, lasR, rhlI,* and *rhlR*), and undertake an analysis of the clonal profile of the isolates.

## METHODS

### Bacterial isolates and their sources

The bacterial isolates analyzed in this study were obtained from patients admitted to a public hospital in Recife, Pernambuco, Brazil. The samples were provided by the bacteriological division of the hospital and were collected between 2018-2019. This study was approved by the Ethics Committee on Research of the Universidade Federal de Pernambuco, Brazil (Ref. No. 0490.0.172.000-11). A total of 31 *P. aeruginosa* isolates were obtained from patients, of which 21 were infection isolates (II), and ten were colonization isolates (CI), based on the criteria adopted by the microbiology laboratory of the hospital. The isolates were stored at -20ºC at the Bacteriology and Molecular Biology Laboratory, Universidade Federal de Pernambuco. The frozen isolates were reactivated in Brain Heart Infusion (BHI) medium, incubated for 24 h at 37ºC, and were subsequently seeded in cetrimide agar and incubated for an additional 24 h at 37ºC. These isolates represented diverse clinical and surveillance samples (Table 1).

**TABLE 1:** Description of *P. aeruginosa* isolates based on the clinical/surveillance sample type.

Identification	Hospital Sector	Source	Classification	Adhesion Profile of Biofilm
**P01**	ICU	Tracheal secretion	Infection	Non-adherent
**P02**	ICU	Rectal swab	Colonization	Weakly adherent
**P03**	ICU	Urine	Infection	Non-adherent
**P04**	ICU	Tracheal secretion	Infection	Weakly adherent
**P05**	ICU	Tracheal secretion	Infection	Weakly adherent
**P06**	ICU	Rectal swab	Colonization	Weakly adherent
**P07**	UCO	Tracheal secretion	Infection	Moderately adherent
**P08**	UCO	Tracheal secretion	Infection	Moderately adherent
**P09**	ICU	Catheter tip	Infection	Weakly adherent
**P12**	CL	Nasal swab	Colonization	Weakly adherent
**P15**	CL	Blood	Infection	Weakly adherent
**P17**	UCO	Rectal swab	Colonization	Non-adherent
**P18**	SC	Urine	Infection	Non-adherent
**P22**	ER	Urine	Infection	Weakly adherent
**P23**	CL	Urine	Infection	Strongly adherent
**P24**	SC	Catheter tip	Infection	Non-adherent
**P27**	UCO	Rectal swab	Colonization	Non-adherent
**P28**	CARDIO	Urine	Infection	Weakly adherent
**P29**	ICU	Blood	Infection	Non-adherent
**P31**	CARDIO	Blood	Infection	Non-adherent
**P32**	CARDIO	Blood	Infection	Non-adherent
**P42**	ICU	Blood	Infection	Non-adherent
**P44**	UCO	Rectal swab	Colonization	Weakly adherent
**P46**	ICU	Catheter tip	Infection	Non-adherent
**P51**	ICU	Rectal swab	Colonization	Weakly adherent
**P59**	UCO	Blood	Infection	Weakly adherent
**P63**	ICU	Rectal swab	Colonization	Weakly adherent
**P66**	UCO	Rectal swab	Colonization	Weakly adherent
**P67**	ICU	Blood	Infection	Weakly adherent
**P70**	UCO	Rectal swab	Colonization	Non-adherent
**P79**	ICU	Catheter tip	Infection	Non-adherent

**ICU:** Intensive Care Unit; **UCO:** Coronary Unit; **CL:** Clinic; **SC:** Surgical Clinic; **CARDIO:** Cardiology Clinic; **ER:** Emergency Room.

### Biofilm production test

*P. aeruginosa* isolates were grown in BHI broth for 24 h at 37ºC. Next, 200 µL of the bacterial suspension was applied to flat bottomed 96-well polystyrene plates in triplicate for microtitration. BHI broth was used as a negative control, and the PA01 strain of *P. aeruginosa* was used as a positive control (CP) to test for biofilm production. The plates were incubated at 37ºC for 24 h, and subsequently, the bacterial suspensions were removed, and each well was washed three times with 250 µL of saline (0.9 % NaCl (Dinâmica)). The bacterial samples were fixed using 200 µL of methanol (Química Moderna) for 15 min. After methanol removal, the plates were left to dry at room temperature and were stained with 200 µL of crystal violet (Newprov) solution for 5 min. 

Next, the plates were washed under running water and dried at room temperature. The absorbance at A_570_ was measured using an ELISA plate reader (BioRad, model 550), and the samples were classified as described by Stepanovic and collaborators (2000)^11^. The optical density for each isolate (ODi) was obtained by averaging the three wells, and this value was compared with the optical density of the negative control (ODc). The isolates were classified into four categories based on the average of the ODi obtained relative to the ODc. The classification criteria for each category included cells that were not adherent (ODi ≤ ODc); weakly adherent (+) (ODc < ODi ≤ 2×ODc); moderately adherent (++) (2×ODc < ODi ≤ 4×ODc); and strongly adherent (4×ODc < ODi).

### Detection of genes related to biofilm production

Total DNA extraction was performed using the Brazol kit (LGC-Biotecnologia), as per the protocol provided by the manufacturer, and the DNA was quantified via spectrophotometry (Ultraspec 3000; Pharmacia Biotech). A simple quantification was performed using the absorbance at 260 nm and an assessment of purity performed using the 260/280 ratio. The genes associated with QS i.e., *lasI, lasR, rhlI*, and *rhlR* ^14^ ^,^ ^15^ were amplified via PCR using the primers listed in Table 2. The parameters used for the PCR were 30 cycles of denaturation at 94ºC for 1 min, annealing at 52ºC for 1 min, and an extension step at 72ºC for 1.5 min. The Blue Green (LGC Biotecnologia, São Paulo) stained PCR products were run on a 2 % agarose gel using electrophoresis and were visualized under UV light. 

**TABLE 2:** Sequences of primers used for the detection of quorum-sensing genes.

Genes	Primers	Base pairs (bp)
***llasI***	5’-CGTGCTCAAGTGTTCAAGG-3’	295
	5’-TACAGTCGGAAAAGCCCAG-3’	
***llasR***	5’-AAGTGGAAAATTGGAGTGGAG-3’	130
	5’-GTAGTTGCCGACGACGATGAAG-3’	
***rrhlI***	5’-TTCATCCTCCTTTAGTCTTCCC-3’	155
	5’-TTCCAGCGATTCAGAGAGC-3’	
***rrhlR***	5’-TGCATTTTATCGATCAGGGC-3’	133
	5’-CACTTCCTTTTCCAGGACG-3’	

### Molecular typing of the isolates

The 31 isolates were analyzed via molecular typing by using the enterobacterial repetitive intergenic consensus-based PCR (ERIC-PCR) technique to determine the clonal profile of the strains. The PCR was performed in a total volume of 25 μL per tube containing 100 ng of DNA, 10 pmol of primers (ERIC-1 [5'-ATGTAAGCTCCTGGGGATTCAC-3']; ERIC-2 [5'AAGTAAGTGACTGGGGTGAGG-3']), 1x buffer, 200 μM deoxyribonucleotide triphosphate (Ludwig Biotec), 1.5mM of MgCl_2_ and 1 U of DNA Taq Polymerase (Promega). The amplification parameters for ERIC-PCR were initial denaturation at 95ºC for 3 min, followed by 30 at 92ºC for 1 min, annealing at 36ºC for 1 min, an extension step at 72ºC for 8 min and a final extension at 72ºC for 16 min. The PCR products were stained with Blue Green (LGC Biotecnologia, São Paulo), and analyzed by agarose gel electrophoresis using a 1.5 % agarose gel. The DNA bands were visualized under UV light and photodocumented (Kasvi) for clonal analysis^21^.

## RESULTS

### Origin of isolates

In the II samples, the highest prevalence of bacterial isolates was detected in blood 33.3 % (7∕21); 23.8 % (5∕21) of the tracheal secretion and urine samples showed the presence of bacteria. In the CI samples, 90 % (9∕10) tested positive, and these were obtained from a rectal swab culture (Table 1).

### Phenotypic analysis of the biofilm production

**FIGURE 1:**
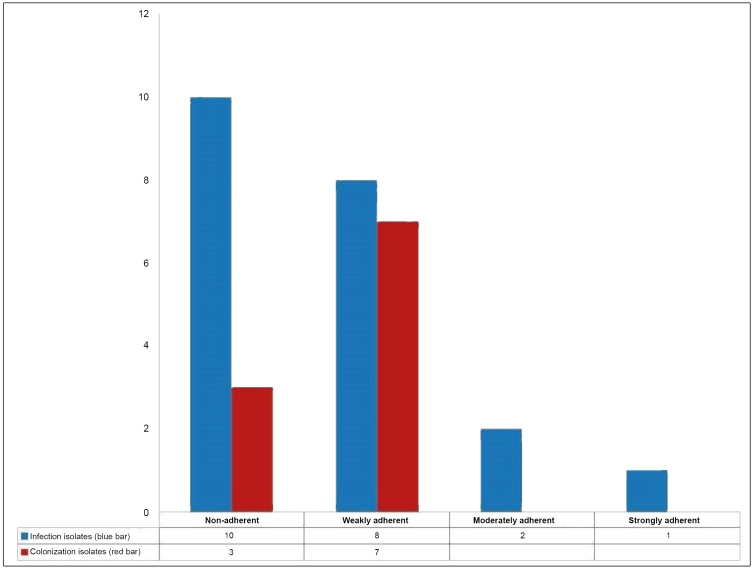
The profile of biofilm production by the infection and colonization isolates.

 Of the 31 isolates studied, 58.1 % (18/31) were biofilm producers. After staining and analysis using the spectrophotometer, the isolates were classified as described in the work by Stepanovic *et al* ^11^. In the CI samples, 70 % (7/10) showed a weakly adherent profile. In the II samples, 52.4 % (11/21) isolates produced biofilms, and these were classified as weak (38.1 %; (8/21)), moderate (9.5 %; (2/21)), and strongly adherent (4.8 %; (1/21)) (Figure 1).

### Quorum-sensing gene analysis

All 31 isolates had the four QS genes studied (*lasI, lasR, rhlI,* and *rhlR*).

### Clonal profiles of isolates

**FIGURE 2:**
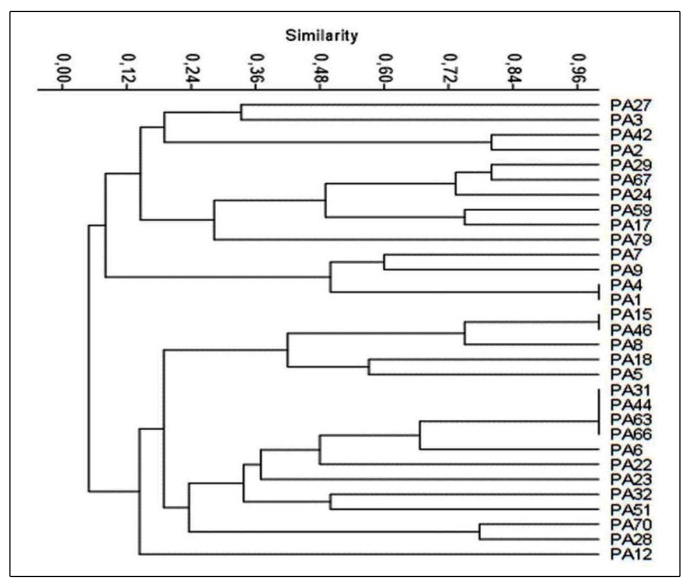
The dendrogram was constructed by analyzing the results of the ERIC-PCR sequences and was generated using the PAST software for 31 clinical isolates of *P. aeruginosa*.

We performed a clonal profile analysis (Figure 2) and identified 26 distinct genetic profiles, that showed high genetic variability between the isolates. Additionally, it is possible to see the occurrence of three clones, one of which composed of four isolates PA31 (infection), PA44, PA63, and PA66 (all of which are colonization isolates). These results indicate that this clone was disseminated between colonized and infected patients.

## DISCUSSION

The II analyzed in this study were obtained from different infection sites, although samples of blood, tracheal secretion, and urine were the most prevalent sources of *P. aeruginosa,* and this data was similar to that from Goncalvez et al., (2017)^22^. The authors described bacteremia without a defined focus of infection as the most frequent source and the respiratory tract as a second site of infection. In a study by Lima et al. (2018)^1^, the authors showed that there was a higher prevalence of infection in samples from tracheal secretions, followed by blood samples. Further, Brusselaers et al. (2011) described the Intensive Care Unit (ICU) of hospitals as showing a high frequency of infections^23^, and *P. aeruginosa* was significantly responsible for a wide range of infections in critically ill patients that were acquired in the ICU.

We used a quantitative phenotypic test considered to be a gold standard to detect biofilm production by clinical isolates of *P. aeruginosa.* Moreover, different studies that used the same technique to analyze biofilm production by *P. aeruginosa* obtained percentages for biofilm production ranging from 73.7 % to 98.6 % in the isolates analyzed^1^ ^,^ ^3^ ^,^ ^6^ ^,^ ^9^ ^,^ ^10^ ^,^ ^24^, that were higher than those described in this study.

The adhesion profile of the biofilm showed a distribution variable of the groups that were classified as weak, moderate, to strongly adherent. Similarly, Perez et al. (2013)^9^ obtained biofilm producer isolates in 93.4 % of the samples tested, and these were further classified as weak (56 %), moderate (24.2 %), and strongly adherent (12.2 %). In a study by Lima et al. (2017)^10^, 75 % of the isolates were biofilm producers, and the authors classified them as weak (40 %), moderate (25 %), and strongly adherent (10 %). Additionally, another study by Lima et al. (2018)^10^ showed 77.5 % of the isolates as biofilm producers, of which 42.5 % were weakly adherent, 27.5 % were moderately adherent, and 7.5 % were strongly adherent. Consequently, these studies showed a predominance of weakly adherent isolates, which corroborates the results of this study.

Biofilm formation is a complex virulence mechanism, which requires gene regulation by different components of the QS system. Changes in the expression of the genes related to the QS network or the presence of mutations can reduce the production of this important bacterial virulence factor^1^ ^,^ ^12^ ^,^ ^17^. The detection of the presence of the QS genes associated with the phenotypic analysis of biofilm production can help in the evaluation of their regulation. In this study, the genotypic analysis of 31 isolates showed a 100 % detection rate for all four investigated genes. Perez et al. (2013)^9^ analyzed 91 isolates of *P. aeruginosa* that were found to infect cystic fibrosis and non-cystic fibrosis patients and demonstrated that all four genes were present in the isolates. Lima et al. (2018)^1^ investigated 40 isolates and described a 100 % detection rate for the *lasR, rhlI,* and *rhlR* genes, and a 97.5 % detection rate for the *lasI* gene. However, these data differ from the results obtained by Karatuna and Yagci (2010)^8^, in which 81.25 %, 68.65 %, and 62.5 % of the isolates were positive for the *lasI* and *lasR* genes, the *rhlI* gene, and the *rhlR* gene, respectively. 

In our study, although all isolates showed the presence of the quorum-sensing genes analyzed, 41.9 % (13∕31) of the isolates did not produce biofilms in the quantitative analysis. This may occur due to the non-expression of these genes or because of the presence of mutations in the genes that regulate the QS system, as found in a study by Senturk et al., (2012)^4^. The authors sequenced the QS genes involved in biofilm production and observed point mutations in the sequences for the *lasR, lasI*, *rhlR*, and *rhlI* genes that prevented their efficient expression. Lima et al., (2018)^1^ identified nine isolates of *P. aeruginosa* that were not biofilm producers. Sequencing of the *lasR* gene showed the occurrence of mutations at position 53 of the LasR protein, which is close to the binding region for its autoinducer, N-(3-oxododecanoyl) homoserine lactone (OdDHL). The authors attributed the non-formation of biofilm by the *P. aeruginosa* isolates to the mutations observed in the *lasR* gene.

Perez et al., (2013)^9^ demonstrated that isolates lacking the *lasR* and *lasI* genes were unable to form a biofilm, and the results indicate that these genes play an important role in QS and the formation of biofilms. These findings were similar to those of Persyn et al., (2019)^25^ who sequenced the genome of 12 *P. aeruginosa* isolates obtained from patients with repeated attacks of pneumonia that were associated with mechanical ventilation. The authors observed that isolates with mutations in the genes associated with the QS system had a lower degree of virulence.

The dendrogram in Figure 2 shows the clustering for the 26 distinct genetic profiles of the isolates obtained, and similar results were observed in other studies^12^ ^,^ ^26^ that evaluated resistance factors in *P. aeruginosa*, such as metalo-β-lactamases, and showed a higher diversity of genetic profiles of *P. aeruginosa* isolates from public hospitals. Additionally, reports from studies in the literature indicate that similar results were obtained using isolates from patients with chronic infections as these individuals are in an environment favorable for the appearance of random mutations. Further, antibiotic therapy promotes the artificial selection of bacteria for greater drug resistance, primarily in cases where *P. aeruginosa* infection is present, as the mucopolysaccharide constituting the biofilm hinders drug diffusion through cell layers^1^ ^,^ ^14^ ^,^ ^17^ ^,^ ^26^.

The analysis of genetic diversity showed the occurrence of a clone composed of four isolates, one of them II and three CI. Johnson et al., (2009)^27^ performed a prospective study for five years and analyzed the colonization of *P. aeruginosa* in patients admitted to the ICU. The results suggest that dissemination between patients plays an important role in the acquisition of *P. aeruginosa* colonization on the skin and mucous membranes, and this process precedes the infection that occurs later. Gómez-Zorrilla et al, (2015)^28^ performed a prospective observational study and identified that the prior rectal colonization by *P. aeruginosa* is a key factor for the development of infection. Additionally, another study^29^ showed that health professionals may directly or indirectly be involved in the microorganism spread chain.

Biofilm production in bacterial isolates is recognized as an important factor for persistent infections^30^. The manifold and diverse mechanisms employed by *P. aeruginosa* to survive antibiotic treatment while growing in a biofilm represent an important therapeutic challenge^31^. Additionally, the acquisition of possible resistance genes is also an important factor^12^; however, this was not analyzed in the present study. Therefore, the emphasis on surveillance culture is important in the implementation of the infection control program, as described by Abdalhamid et al*.* 2016^32^ in a study carried out in ICU patients.

## CONCLUSIONS

In this study, we described the biofilm production in infection and colonization isolates of *P. aeruginosa* from patients admitted to a hospital. Additionally, we detected the presence of high genetic diversity between the isolates, including a clone composed of among infection and colonization isolates, indicating that dissemination had occurred in the hospital environment. The results indicate the importance of the detection of biofilms in *P. aeruginosa* in both colonization and infection isolates, especially from complex environments such as ICUs. The strategy outlined in this study can be used for monitoring and studying strains that can cause infections in hospitalized patients, though the formation of biofilms remains an important therapeutic challenge. 
